# Supplementary material for: Asynchronous magnetic resonance elastography: Shear wave speed reconstruction using noise correlation of incoherent waves
Source: Magn Reson Med. 2022 Oct 27;89(3):990–1001. doi: 10.1002/mrm.29502 (PMC9792433; doi:10.1002/mrm.29502)
Supplement: Supplementary file 1 — DATA S1 MATLAB code used to generate the numerical simulations of Figure 1 [file MRM-89-990-s002.zip › k-Wave/helpfiles/gaussian.html]

gaussian :: Functions (k-Wave)


# gaussian

Create a Gaussian distribution.

## Syntax

```
gauss_distr = gaussian(x)
gauss_distr = gaussian(x, magnitude)
gauss_distr = gaussian(x, magnitude, mean)
gauss_distr = gaussian(x, magnitude, mean, variance)
```

## Description

`gaussian` returns a Gaussian distribution f(x) with the specified magnitude, mean, and variance. If these values are not specified, the magnitude is normalised and values of variance = 1 and mean = 0 are used. For example running

```
x = -3:0.05:3;
plot(x, gaussian(x));
```

will plot a normalised Gaussian distribution.

Note, the full width at half maximum of the resulting distribution can be calculated by `FWHM = 2 * sqrt(2 * log(2) * variance)`.

## Inputs

|  |  |
| --- | --- |
| `x` | x-axis variable |

## Optional Inputs

|  |  |
| --- | --- |
| `magnitude` | bell height (default = normalised) |
| `mean` | mean or expected value (default = 0) |
| `variance` | variance ~ bell width (default = 1) |

## Outputs

|  |  |
| --- | --- |
| `gauss_distr` | Gaussian distribution |

## See Also

`getWin`
